# Investigating Confined Space Asphyxias: Plastic Bag Involvement and Gas Inhalation – A Case Series and Literature Review

**DOI:** 10.15388/Amed.2024.31.2.10

**Published:** 2024-12-04

**Authors:** Sofija Saulė Kaubrytė, Sigitas Chmieliauskas, Giedrė Kažukauskė, Sigitas Laima, Diana Vasiljevaitė, Jurgita Stasiūnienė

**Affiliations:** 1 Faculty of Medicine, Vilnius University, Vilnius, Lithuania; 2 Department of Pathology, Forensic Medicine, Institute of Biomedical Sciences, Faculty of Medicine, Vilnius University, Vilnius, Lithuania

**Keywords:** Asphyxia, confined space, plastic bag, suicide, autopsy, forensic medicine, Raktažodžiai: asfiksija, uždusimas uždaroje erdvėje, asfiksija plastikiniu maišeliu, savižudybė, autopsija, teismo medicina

## Abstract

**Background:**

Asphyxia within confined spaces, particularly involving plastic bag suffocation and gas inhalation, presents complex forensic challenges. This study explores mechanisms, epidemiology, and forensic considerations of these fatalities, drawing from a comprehensive literature review and analysis of four clinical cases. Plastic bag asphyxia, exacerbated by oxygen depletion and carbon dioxide accumulation, induces rapid loss of consciousness and cardiorespiratory arrest. The method’s lethality is increased when combined with inert gases like helium, known for their rapid onset of hypoxia and painless nature. Recent research demonstrates a growing incidence of plastic bag suffocation as a method of suicide. The accessibility of suicide-related information on online platforms contributes to the dissemination of new methods, posing challenges for suicide prevention efforts. Forensic investigations often face obstacles in accurately determining the cause and manner of death, with nonspecific autopsy findings and scene manipulations complicating the diagnostic process.

**Materials and methods:**

A literature research was conducted across PubMed and Google Scholar databases, focusing on articles published in the last 5 years, applying keywords relevant to the topic under consideration and their combinations. 34 pertinent articles were selected, supplemented by data from the Lithuanian State Forensic Medicine Service, involving four clinical cases of confined space asphyxia. Autopsy findings, toxicological analyses, and contextual details were thoroughly examined to determine the mechanism and circumstances of death.

**Results:**

Plastic bag suffocation, often combined with inert gas inhalation, emerges as a common method in suicides. Demographic analyses reveal distinct patterns, with younger age groups demonstrating an increased tendency for gas inhalation suicides. The accessibility and perceived painlessness of helium contribute to its rising usage for suicide purposes. Forensic challenges include nonspecific autopsy findings, manipulations of death scenes, and difficulties in detecting inert gases postmortem. Enhanced surveillance and efforts to restrict access to these tools are crucial in preventing the spread of new suicide methods.

**Conclusions:**

The study highlights the multifaceted nature of asphyxiation deaths within confined spaces and the importance of proactive interventions in suicide prevention. Enhanced surveillance, tailored prevention strategies, and collaborative efforts are essential in addressing evolving suicide methods and reducing their impact.

## Abstract (it)

**Įvadas:**

Asfiksija uždaroje erdvėje, savižudžio galvai esant plastikiniame maišelyje, yra nulemta sumažėjusios deguonies koncentracijos aplinkos įkvepiamame ore bei gali būti dėl burnos ir nosies obturacijos. Vertinančiam asfiksijos uždaroje erdvėje atvejus teismo medicinos ekspertui gali kilti sunkumų nustatyti mirties priežastį, nes daugeliu atvejų matomi nespecifiniai staigios asfiksinės mirties požymiai, o iš įvykio vietos mirusiojo kūnas dažnai pristatomas nuėmus maišelį nuo galvos. Autopsijos metu gali būti matomos taškinės kraujosruvos akių junginėse, veninė vidaus organų pilnakraujystė, taškinės kraujosruvos širdies ir plaučių paviršiuose, plaučių bei galvos smegenų pilnakraujystė bei edema. Histologinio tyrimo metu paprastai taip pat gali būti nustatomi bendri, nespecifiniai staigios asfikcinės mirties požymiai. Šiame tyrime nagrinėjami šios mirties mechanizmas, epidemiologija ir teismo medicinos ekspertizių duomenys, remiantis išsamia literatūros apžvalga ir keturių klinikinių atvejų analize.

**Metodai:**

Pagal nagrinėjamą temą apibūdinančius raktinius žodžius ir jų derinius buvo atlikta 5 metų laikotarpiu publikuotų straipsnių paieška *PubMed* duomenų bazėje ir internetinėje *Google Scholar* paieškos sistemoje. Literatūros apžvalgai buvo atrinkti 34 straipsniai, išspausdinti anglų kalba. Remiantis Valstybinės teismo medicinos tarnybos pateiktais duomenimis, analizuoti 4 klinikiniai atvejai ir aprašyti asfiksijos uždaroje erdvėje mechanizmą iliustruojantys autopsijų radiniai. Išsamiai vertintos autopsijų išvados, toksikologinės analizės, nurodyti dažniausiai įvykio vietoje aptinkami susiję įkalčiai bei kitos svarbios asfiksijų uždarose erdvėse nulemtos mirties aplinkybės.

**Rezultatai:**

Plastikinio maišelio asfiksija, dažnai kartu įkvepiant inertinių dujų, yra aprašoma kaip visuomenėje plintantis savižudybės metodas. Stebima tendencija, jog helio dujas savižudybės tikslu dažniau pasitelkia jaunesni asmenys. Teismo medicinos ekspertizių metu iškyla atitinkamų iššūkių: asfiksijų plastikiniu maišeliu atvejais nustatomi nespecifiniai autopsijos radiniai, svarstomos prielaidos dėl kitų asmenų atliktų manipuliacijų lavoną supančioje aplinkoje, taip pat susiduriama su diagnostiniais sunkumais, siekiant aptikti inertines dujas mirusiojo organizme.

**Išvados:**

Asfiksijos uždaroje erdvėje išsivysto dėl deguonies išeikvojimo ir anglies dioksido koncentracijos padidėjimo uždaroje aplinkoje, visa tai lemia greitą sąmonės netekimą ir mirtį. Teismo medicinos gydytojai nustato mirčių dėl asfiksijų uždarose erdvėse atvejus remdamiesi ne tik autopsijos bei papildomų tyrimų duomenimis, bet ir įvertina svarbias tyrimui mirusiojo radimo aplinkybes. Išsiaiškinti mirties priežastį padeda detali įvykio vietos apžiūra, atsižvelgiama į lavono kūno padėtį, vertinami pastebėti pašaliniai daiktai ant mirusiojo kūno bei šalia esančioje aplinkoje.

## Introduction

Asphyxiation occurring within a confined environment, focusing on those cases when victim’s head is placed in a plastic bag, is frequently attributed to the depletion of oxygen within the inhaled air, often compounded by obstruction of the oral and nasal passages. Forensic medicine doctors and pathologists encounter significant challenges in determining the cause of death in such cases. Two of the most frequently occuring situations that mislead specialists are victim’s body replacement from its original place and removal of plastic bag from victim’s head. Autopsy examinations of such cases typically reveal nonspecific pathological findings, including petechial hemorrhages in the conjunctiva of the eyes, surfaces of the heart and lungs, venous congestion of internal organs, pulmonary emphysema and cerebral edema. Furthermore, histological analyses commonly unveil additional nonspecific markers indicative of sudden asphyxiating deaths, with particular indicators like Hypoxia Induced Factor 1-alpha (HIF-1α) and Surfactant Protein A (SP-A) serving as valuable aids in confirming asphyxia-related fatalities. The purpose of this study is to conduct a review of the current literature associated to asphyxiation in confined spaces, specifically focusing on cases involving plastic bag asphyxia. Additionally, this research aims to present and analyze four detailed case reports of individuals who died due to asphyxiation within confined spaces, emphasizing the significance of precise scene examination in detecting the circumstances surrounding each fatality. By integrating case observations with existing literature, this study attempts to enhance the understanding and awareness of the complexities associated with asphyxiation in confined environments, ultimately contributing to improved investigation practices and preventive measures.

## Methods

A circumstantial search was conducted across the PubMed database and the online Google Scholar search engine using keywords relevant to the subject, including “asphyxia,” “suffocation,” “closed environment,” “confined space,” “plastic bag,” “gas inhalation,” and “suicide.” The search involved articles published in English language within a five-year timeframe (papers from 2019 up to 2024 were screened), selecting a total of 34 pertinent articles for inclusion in the literature review. Additionally, associated information to the analysis of four clinical cases, sourced from the Lithuanian State Forensic Medicine Service, has been involved in the examination. Autopsy findings from these cases were examined to clarify the mechanisms of asphyxia within a closed space. Detailed information on each case was provided by including essential details such as incident location, time of death, and the presumed cause of death. A full body autopsy was performed on all deceased individuals to thoroughly examine the circumstances surrounding their death. During the autopsy, blood and urine samples were systematically collected for alcohol, medications and drug testing.

## Practical examples

### Case 1

During the investigation of a deceased 24-year-old male, who was found in his room, it was determined that the victim had been asphyxiated by helium gas. The examination of the body revealed intentional staging: a black polyethylene bag secured tightly around the neck with gray adhesive film, a white-transparent tube which connected to a helium gas cylinder was placed inside the bag. The victim was found reclining on a bed.

**Fig. 1:**
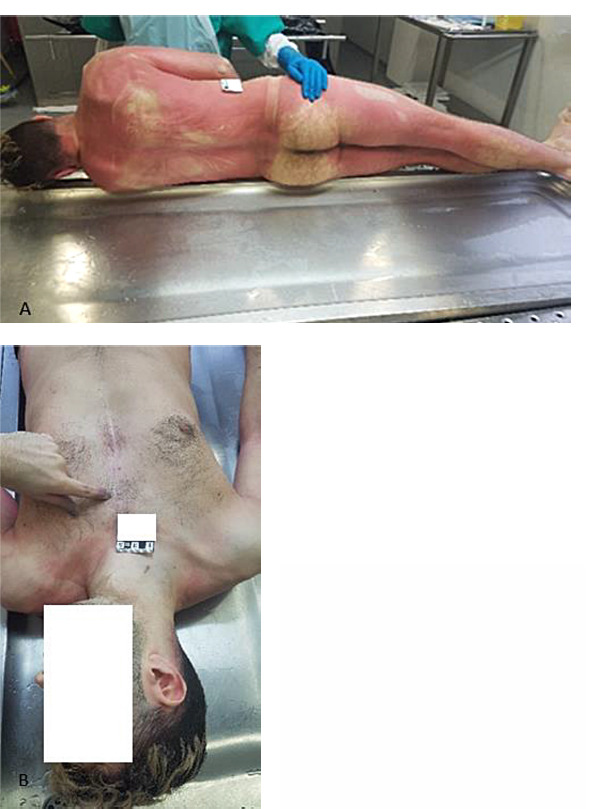
The image (a) presents a detailed view of the dorsal aspect of a cadaveric male specimen, highlighting the pronounced livor mortis, a characteristic manifestation of post-mortem vascular changes, with evident discoloration discernible beneath the integumentary surface. In (b), a distinct scar is visible on the chest area, serving as a focal point of external examination and potentially indicating antemortem surgical interventions or traumatic incidents.

Although a chest scar was noted during external inspection, no other signs of trauma were found (Fig. 1b). However, internal examination revealed nonspecific signs of asphyxiation, including petechial hemorrhages in the conjunctiva of the eyes and on the surfaces of the heart and lungs. Additionally, the presence of unclotted blood within chambers of the heart, venous congestion of internal organs, and cerebral and pulmonary edema emphasized the severity of the asphyxiation.

Toxicological analysis of the deceased’s blood detected concentrations of quetiapine (0.020 mg/l), citalopram (0.021 mg/l), and bromazepam (0.092 mg/l), suggesting prior ingestion of these substances. Furthermore, metabolites such as alpha-hydroxyalprazolam and 3-hydroxybromazepam were discovered in the urine, along with trace amounts of bromazepam, quetiapine, citalopram, and mirtazapine. No ethyl alcohol was found in both blood and urine samples.

Based on all of the findings during the investigation of a deceased 24-year-old man, including the absence of ethyl alcohol in both blood and urine samples, the circumstances surrounding the victim’s death, the presence of petechial hemorrhages on vital organs, internal organ congestion, and cerebral and pulmonary edema, it was concluded that the cause of death was confined space asphyxiation.

### Case 2

**Fig. 2:**
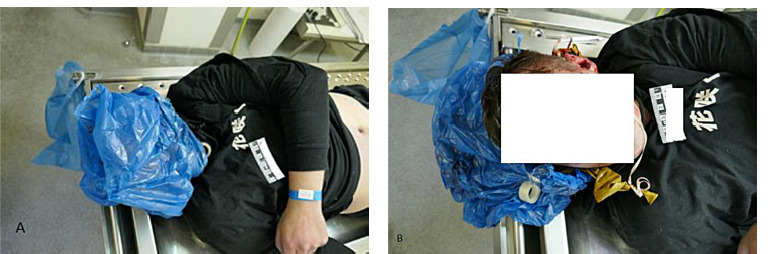
(a) The victim’s head is enveloped by a blue plastic bag, tightly sealed with adhesive tape. (b) Upon detailed external inspection, within the bag enveloping the deceased, a burst golden balloon was observed alongside three wounds on the nose, each displaying irregular edges and surrounded by reddish-purple bruises.

The examination of the deceased, a 27-year-old man, provided crucial insights into the circumstances of his death. The individual was discovered in his living room, lying face down on the floor, with no signs of visible physical harm. A blue plastic bag covered victim’s head and was tightly sealed with an adhesive tape (Fig. 2a). In the bag a burst golden balloon was detected.

However, upon the further examination, during the detailed external inspection three separate wounds were detected. All three wounds, each measuring exactly 0.5 cm in diameter, with irregular edges and surrounded by a reddish-purple bruise approximately 1 cm in diameter were located on the deseased’s nose (Fig. 2b). During the internal autopsy, although the structures of the throat and neck bones were intact, noticeable petechial hemorrhages were observed on the conjunctiva, epiglottis, and surfaces of the heart and lungs. Additionally, dark, unclotted blood was found in the heart chambers, and significant swelling was observed in the brain and lungs.

Subsequent toxicological analysis revealed ethyl alcohol concentrations of 1.51‰ (per mille) in the blood and 2.36‰ (per mille) in the urine, offering insights into the deceased’s condition before death. Ultimately, the combination of external and internal findings, along with toxicological results, indicated the suffocation in a confined space as the cause of death. All of these evidences shed light on tragic circumstances surrounding the victim’s death, providing a clarity to the investigation.

### Case 3

**Fig. 3:**
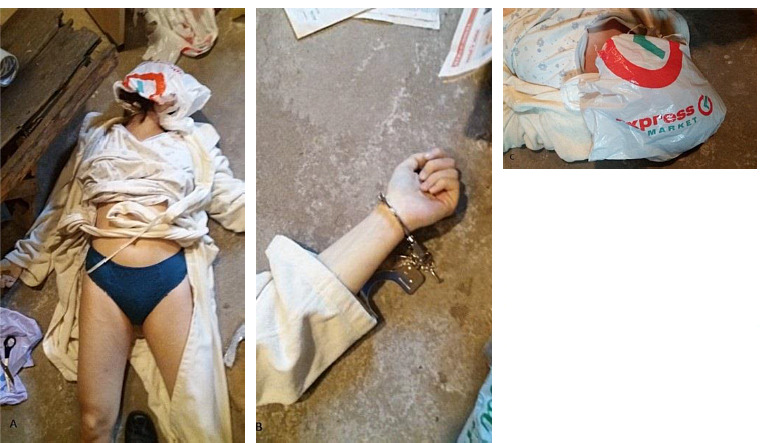
(a) Evidence of previous self-harm was identified on the victim’s body, including numerous scars present on the thighs. (b) A pair of handcuffs encircled the left wrist of the deceased woman. (c) Several plastic bags were tightly wrapped around the deceased woman’s head and sealed with adhesive tape.

The deceased, 24-year-old woman, was discovered in the confined basement. There were suspicions of suicide, substantiated by reports of prior suicide attempts due to her struggles with depression. Noteworthy findings during the external examination included the presence of handcuffs on her left wrist, as well as three plastic bags tightly sealed around her head with an adhesive tape (Fig. 3b, 3c). Additionally, the presence of prior self-harm was detected on victim’s body, such as multiple scars on the thighs (Fig. 3a).

**Fig. 4:**
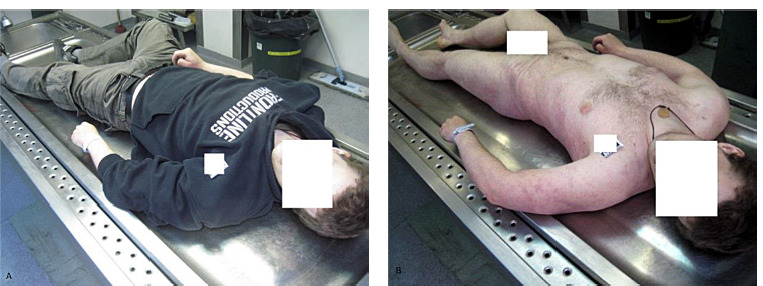
(a), (b) Images of a male cadaver lying in a supine position. The plastic bag, previously enveloping the victim’s head, was removed prior to the onset of the external examination.

Further autopsy examination unveiled nonspecific findings consistent with asphyxiation-induced death, such as unclotted blood within heart chambers, venous congestion within internal organs, and evident edema observed in both lungs and brain. A detailed toxicological evaluation of victim’s blood and urine showed no traces of ethyl alcohol, illegal substances or medications. In conclution, all of these findings suggested confined space asphyxiation.

### Case 4

Upon conducting a thorough examination of a deceased male, aged 25, who was discovered in a basement, several significant observations emerged. The body was found in a supine position. Notably, a transparent plastic bag with a tube in it and connected to a gas cylinder, was detected on the head of the deceased.

External inspection revealed nonspecific findings. Victim’s skin presented a pale yellowish color, with purple livor mortis on the dorsal surfaces. Rigor mortis was notably pronounced across all muscle groups. No external injuries were detected on victim’s body. Subsequent internal examination supported the suspicion of asphyxiation. The lungs and brain showed signs of edema, alongside venous congestion of internal organs. Further investigation revealed no evidence of trauma or pathological anomalies within the cranial or thoracic cavities. Toxicological analysis showed negative results for ethyl alcohol, medications and illegal substances in both blood and urine samples.

Summarizing autopsy findings and the circumstances of victim’s discovery, the cause of death was determined as suffocation within an enclosed environment.

The findings of all 4 cases with determined confined space asphyxia are summarized in Table 1.

**Table 1:** 4 cases, where death occurred as a result of asphyxiation in confined spaces.

	Case 1	Case 2	Case 3	Case 4
*Gender*	Male	Male	Female	Male
*Age*	24	27	24	25
*Place*	Room	Room	Residential basement	Residential basement
*Lethal tools*	Plastic bag + adhesive tape + connection tube + helium gas cylinder	Plastic bag + adhesive tape + burst balloon within	Several plastic bags (×3) + adhesive tape	Plastic bag + adhesive tape + connection tube + gas cylinder
*Alcohol in blood (*‰)	Not detected	1.51	Not detected	Not detected
*Alcohol in urine (*‰)	Not detected	2.36	Not detected	Not detected
*Traces of psychoactive substances and other drugs in blood/urine*	quetiapine, citalopram, bromazepam, mirtazapine, metabolites (alpha-hydro-xyalprazolam, 3-hydro-xybromazepam)	Not detected	Not detected	Not detected
*Autopsy findings*	scar on a chest petechial hemorrhages (conjunctiva of the eyes, surfaces of the heart and lungs) unclotted blood within heart chambers internal organ congestion cerebral and pulmonary edema	three separate wounds (nose) petechial hemorrhages (conjunctiva, epiglottis, surfaces of the heart and lungs) unclotted blood within heart chambers cerebral and pulmonary edema	multiple scars (thighs) unclotted blood within heart chambers internal organ congestion cerebral and pulmonary edema	purple livor mortis on the dorsal surfaces rigor mortis across all muscle groups internal organ congestion cerebral and pulmonary edema

## Discussion

Confinement in small spaces can lead to a deadly deprivation of oxygen, termed as confined space asphyxia [1]. Plastic bag asphyxia, a subtype of confined space asphyxia, involves a combination of oxygen depletion and an increase in carbon dioxide concentration [2,3,4]. This suffocation mechanism induces rapid loss of consciousness, potentially leading to a sleep state followed by coma and irreversible cardiorespiratory arrest [2,5]. The physical obstruction caused by the plastic bag put over the head, along with oxygen depletion and possible stimulation of sympathetic nervous system, initiates a rapid cardio-inhibitory process, including arrhythmias and ventricular fibrillation [2,5,6]. Helium, frequently utilized in conjunction with plastic bags for suicide purposes, acts as an inert gas displacing oxygen, leading to asphyxia [4,7–9]. Its rapid removal of oxygen from the lungs accelerates the onset of hypoxia and loss of consciousness, typically within seconds [4,7,8,10]. Unlike other asphyxiation methods, helium inhalation does not trigger the breathing reflex, making it painless and attractive for suicide purposes [4,7,8,10]. Moreover, inert gases like helium may cause direct injuries to the pulmonary interstitium, leading to respiratory dysfunction and fatal air embolism [3,7]. Studies also suggest that prolonged inhalation of helium can lead to hypoxic-ischemic encephalopathy and irreversible brain damage within minutes [4,11,12]. Furthermore, helium inhalation can cause a decrease in oxygen concentration in the blood, potentially inducing lethal hypoxia [4,7].

The usage of plastic bags as a means of asphyxiation has drawn significant attention within forensic medicine due to its association with various manners of death. Studies have documented cases of plastic bag suffocation in instances of homicide, suicide, and unintentional fatalities, with a notable increase observed in suicide and accidental deaths [13–15]. Plastic bag suffocation, often combined with other methods such as inhalation of volatile compounds or pharmacological substances, has been increasingly reported as a suicide method over the past few decades [9,13–16].

The advent of single-use plastic carrier bags in the late 1950s marked the onset of deaths related to plastic sheets, often involving inadvertent asphyxiation, especially among children who inserted their heads into the bags [14]. Plastic bag suffocation remains a significant concern, particularly in developed countries, despite its relatively low prevalence compared to other methods of suicide [13,14]. Additionally, research from regions such as Colombia sheds light on the widespread occurrence of plastic bag suffocation in homicidal scenarios, particularly affecting young males [17]. This underscores the complexity of the issue, highlighting not only its role in suicides but also its grim presence in cases of homicide. Moreover, plastic bag suffocation has been associated with various circumstances, including complex suicides, autoerotic asphyxia, confined space asphyxia in children, and suicide pacts [2,3,16,18–20]. Complex suicides, involving multiple methods, have become increasingly documented, showcasing a shift in suicidal behavior. Plastic bag suffocation often serves as a primary method in these cases, particularly when combined with inert gas inhalation [1,3,16,21]. The association between plastic bag suffocation and inert gas inhalation is noted to be synergistic, contributing to the lethality of the method [3]. Furthermore, planned complex suicides involving plastic bag suffocation and gas inhalation highlight deliberate intentions and premeditation by the individuals [1]. Autoerotic asphyxia, a niche within the spectrum of asphyxiation deaths, also involves plastic bag suffocation, often in conjunction with sexual activity and volatile gas inhalation, highlighting the multifaceted nature of plastic bag suffocation within the spectrum of asphyxiation deaths and the diverse contexts in which this method is employed [18].

Demographic analyses reveal distinct patterns associated with different suicide methods. For instance, gas inhalation methods, including inert gases such as helium and nitrogen, have been linked to individuals with prior contact with euthanasia societies, access to instructional materials, and online purchases of gases [22]. Furthermore, younger age groups, including individuals under 25 years, exhibit a heightened propensity for helium and nitrogen suicides, possibly due to increased awareness facilitated by online resources [22]. The emergence of suicide-related websites has been implicated in influencing adolescents’ suicidal behaviors [12]. The proliferation of such online resources, particularly in regions like Japan, has raised concerns about their role in disseminating information on suicide methods [12]. Notably, these platforms may contribute to the accessibility of suicide tools and methodologies, potentially influencing the choice of means among vulnerable individuals, including adolescents [9,11,12,22].

The application of inert gas inhalation as a method for suicide has garnered notable attention, particularly since the early 2000s with the dissemination of instructions pertaining to the use of helium in conjunction with a plastic bag [2–4,9–11,23,24]. Helium, once relatively uncommon, witnessed a substantial increase following its endorsement in the “Final Exit” manual, leading to a surge in suicide deaths by helium asphyxiation, particularly in the USA [2,4,9,11,23,24]. Similarly, the prevalence of helium and nitrogen suicide deaths has risen globally, with notable increases reported in Australia, the UK, the US, and Hong Kong [4,9,10,19,22–24,33]. In Milan, Italy, a forensic analysis revealed that plastic bag suffocation combined with inert gas inhalation was the most prevalent method among complex suicides, constituting 24.5% of cases. This synergistic association, particularly with butane gas, has been increasingly observed, notably in jail settings [3,25]. Similarly, in Friuli, Italy, Simonit et al. identified plastic bag suffocation as a common method, often associated with volatile compound inhalation, indicating a widespread trend. The availability of detailed information on websites and social networks has contributed to the dissemination of this method [26].

The method of suicide by plastic bag asphyxia, often combined with the ingestion of pharmacological substances, presents a complex interplay of psychological, pharmacological, and environmental factors. Gentile et al. (2021) highlight the frequent association of plastic bag suffocation with the ingestion of benzodiazepines and antidepressants, suggesting a synergistic effect between the pharmacological substances and asphyxiation [3]. This observation aligns with the findings of Byard et al., who noted toxic or lethal levels of prescription medications in a significant proportion of cases, with benzodiazepines being the most frequently encountered drugs [2]. Furthermore, Murakami et al. (2019) reported the detection of sedative drugs such as alcohol, benzodiazepines, and antidepressants in the blood and/or urine samples of individuals who committed suicide by plastic bag suffocation, indicating the role of these substances in mitigating the distress associated with asphyxiation [12]. The choice of plastic bag asphyxia as a suicide method may also be influenced by perceptions of efficacy and minimal discomfort. Shields and Hunsaker (2020) note Derek Humphry’s recommendation, as outlined in his book “Final Exit,” to take sleeping pills or tranquilizers prior to the act, potentially to alleviate anxiety and discomfort associated with the method. A significant increase in suicides by plastic bag asphyxia was observed following the publication of materials like “Final Exit,” which may have influenced individuals with psychiatric disorders to adopt this method [2]. Moreover, the context in which plastic bag asphyxia is employed, such as imprisonment, adds another layer of complexity. Gentile et al. (2021) report cases of inmates using everyday objects like plastic bags as a means of suicide, underscoring the accessibility and perceived safety of such methods within the prison environment [25]. Additionally, the association between plastic bag asphyxia and confined spaces highlights the concomitant risk factors, including physical illness, advanced age, and media influence on suicide reporting [23]. Alternative methods, such as suicide by gas inhalation, have also emerged, with variations like helium inhalation being adapted to minimize discomfort and facilitate a peaceful death [22]. The ease of access to helium and nitrogen gases may particularly appeal to individuals with perceived low quality of life due to health or social reasons [15,22].

Helium, an inert gas, has garnered attention as a method of suicide due to its availability, ease of access, and perceived painlessness. The literature indicates a rise in suicides using inert gases such as helium, argon, and nitrogen. While inert gases are generally considered safe, they pose a risk of simple asphyxiation when inhaled in sufficient quantities [3,4,6,8,9–11,27]. The physical properties of helium contribute to its suitability for suicide. Being odorless, colorless, and nonflammable, helium is difficult to detect using standard toxicological analyses, presenting challenges for forensic investigations [8,10]. Furthermore, its rapid dispersal in ambient air and low solubility in blood and tissues make postmortem detection challenging [6,28]. Several methods have been proposed for detecting helium in biological samples, primarily using gas chromatography coupled with mass spectrometry (GC-MS) or thermal conductivity detection (GC-TCD). GC-MS offers high sensitivity and selectivity, with hydrogen as the preferred carrier gas for optimal results [6,8,10,28,32]. However, the selection of appropriate biological samples is crucial for accurate detection. Lung biopsies, airway samples, brain tissue, and cardiac blood are recommended matrices for helium detection, with lung samples being particularly informative due to helium’s pathophysiology [4,6,8,10,28]. Studies have highlighted the importance of proper sampling techniques and storage conditions to minimize helium loss and ensure accurate analyses [6,10,11]. Immediate collection of samples in sealed containers and prompt analysis are recommended to mitigate the risk of gas diffusion and contamination [29]. Additionally, the use of internal standards, such as neon-21, can aid in quantitative helium analysis and minimize methodological challenges [4,8]. Despite advancements in detection methods, challenges persist in accurately determining helium exposure postmortem. Variability in sampling techniques, analytical methods, and the selection of suitable matrices contribute to inconsistencies in helium detection [4,6,8,10,28]. Moreover, the lack of standardized protocols and international guidelines further complicates forensic investigations involving inert gas-related deaths [27,28,30].

The literature on forensic pathology concerning deaths related to confined space asphyxia, particularly those involving plastic bag suffocation and gas inhalation, underscores the complexities in accurately determining the cause and manner of death. Several studies have highlighted the challenges faced by forensic experts in conducting thorough investigations and making precise diagnoses in such cases. A comprehensive examination of the findings reveals recurring themes across the studies. Autopsy findings consistently point to nonspecific signs of asphyxia, including facial congestion, petechiae in the conjunctivae and skin, and pulmonary edema [2,5,6,14–16,20,25,31]. Histological analyses often fail to yield specific indicators of the cause of death, further complicating the diagnostic process [2,5,6,10,14–16]. Specific markers such as Hypoxia Induced Factor 1-alpha (HIF-1α) and Surfactant Protein A (SP-A) can aid in confirming asphyxia-related deaths [14]. However, the absence of characteristic macroscopic and microscopic postmortem changes in certain methods of suicide, such as helium inhalation, poses significant challenges for forensic pathologists [2,6,10,14]. The significance of a meticulous examination of the death scene cannot be overstated. Manipulation of the scene, whether to conceal evidence of autoerotic activities, homicides, or suicides, can obscure vital clues necessary for accurate determination of the circumstances surrounding death [2,6,9,10,14–16,30]. Moreover, societal stigma associated with certain suffocation methods may influence scene presentation and reporting [2,5,16]. The presence or absence of specific items, such as plastic bags, gas tanks, or literature describing suicide methods, can provide valuable insights into the events leading to death [2,6,14–16]. However, even when evidence is present, interpretation remains complex. Psychological autopsies may offer insights into the psychological factors contributing to the death, while extensive toxicological analysis can help identify potential volatile substances or inert gases involved in the fatal event [2,10,15,25,34].

In recent literature on suicide prevention measures, several studies offer insights into the evolving landscape of suicide methods and stress the importance of proactive interventions. Van den Hondel et al. [9] delve into helium inhalation as a method of suicide in the Netherlands, revealing a persistent prevalence despite efforts to introduce preventive measures. In contrast, Sinyor et al. [24] illuminate emerging methods of suicide by gas inhalation in Toronto, highlighting the need for enhanced surveillance, means restriction, and timely interventions to curb their dissemination. Additionally, Burnett et al. [22] underscore shifting trends in gas usage for suicide attempts in Australia, signaling the necessity for dynamic adjustments in prevention strategies to address evolving methods.

These studies collectively emphasize the imperative of proactive measures in suicide prevention. Firstly, timely surveillance is paramount in identifying emerging methods and promptly implementing targeted interventions. Secondly, means restriction efforts, such as limiting access to specific materials or enhancing safety features in their distribution, are crucial in deterring individuals from employing lethal methods. Furthermore, engagement with media and internet platforms is pivotal in mitigating the social contagion effect associated with novel suicide methods, thereby preventing their widespread adoption. Importantly, these findings underscore the need for gender-sensitive approaches, as men are disproportionately affected by certain suicide methods. Engaging men in care and providing tailored support services are vital components of comprehensive suicide prevention strategies. In the Netherlands, despite the preparation of helium tanks with additional oxygen by manufacturers, the prevalence of helium method suicides has not declined, in contrast to observations in other countries such as Australia [9,22]. This suggests that preventive measures, akin to those possibly implemented in Australia, may not have been as effective in the Dutch context. In Hong Kong, initiatives aimed at preventing suicides by helium inhalation, including the introduction of helium tanks filled with extra oxygen, have been documented [9]. This underscores the importance of proactive public health strategies tailored to specific cultural and regional contexts. The study by Sinyor et al. [24] highlights the emergence of novel methods of suicide by inhalational asphyxia in Toronto, mirroring trends observed globally. The findings underscore the need for heightened surveillance of such deaths and targeted efforts to restrict access to compressed gases and charcoal, which are commonly employed in these methods. Moreover, the study emphasizes the importance of timely intervention and media engagement to mitigate the spread of information regarding novel suicide methods, which can contribute to social contagion. In Australia, Burnett et al. [22] emphasize the necessity of vigilant monitoring of changing trends in gas usage for suicide attempts. The study suggests that means restriction efforts may need to be recalibrated to address the uptake of alternative gases, potentially more accessible than those currently targeted by prevention measures. This underscores the dynamic nature of suicide methods and the ongoing need for adaptive prevention strategies. Effective strategies must encompass surveillance, means restriction, timely intervention, and targeted outreach, taking into account regional variations and emerging trends in suicide methods. Moreover, collaboration between researchers, policymakers, and stakeholders is essential to formulate and implement evidence-based interventions aimed at reducing suicide rates and mitigating the impact of novel suicide methods [9,22,24].

In our conducted study involving four individual cases of asphyxiation within confined spaces, notable findings emerge, elucidating the polyvalent nature of these tragic incidents. In *case 1*, a 24-year-old male was found with a helium-filled plastic bag tightly secured around his neck, leading to nonspecific signs of asphyxiation upon examination, including venous congestion, cerebral and pulmonary edema. Toxicological analysis revealed the presence of psychoactive substances, further complicating the forensic assessment. Similarly, *case 2* demonstrated a 27-year-old male discovered with a blue plastic bag covering his head, alongside nasal wounds and internal injuries consistent with asphyxiation, exacerbated by ethyl alcohol intoxication. Conversely, *case 3* involved a 24-year-old woman, suspected of self-inflicted asphyxiation due to depressive struggles, evidenced by multiple plastic bags secured over her head and the absence of toxic substances, further supporting the determination of suicide. Finally, *case 4* reveals a 25-year-old male found with a plastic bag connected to a gas cylinder, exhibiting postmortem hypostasis and internal signs of asphyxiation, with toxicological analysis submitting negative results. Across the cases, commonalities such as the presence of plastic bags tightly sealed around the victims’ heads and nonspecific autopsy findings, including venous congestion and pulmonary edema, which are compatible with asphyxiation as the cause of death, underscore the lethality of such circumstances. However, differences in circumstances, including the presence of psychoactive substances in some cases and indications of self-inflicted harm in others, highlight the diverse pathways leading to death in these scenarios. While all cases underscore lethal consequences of asphyxiation within confined spaces, findings in presentation and contributory factors, such as substance ingestion and mental health history, highlight diverse pathways leading to death in these scenarios. This comprehensive examination sheds light on the complexities inherent in forensic investigations of asphyxiation-related fatalities, emphasizing the importance of thorough examination and consideration of individual findings in determining the cause of death.

## Conclusions

Asphyxiation within a confined space, particularly plastic bag asphyxia, occurs as a consequence of oxygen depletion and carbon dioxide accumulation, accelerating loss of consciousness and ultimately leading to fatality. Forensic investigators play a fundamental role in investigating those rare cases of plastic bag asphyxiation. Thorough collection of evidences from the scene, knowledge of detailed victim’s medical history and autopsy findings play the most important role in determining a cause of death. During the external examination of a deceased, specialists must pay attention to details such as presence of a plastic bag on victim’s head and devices such as helium gas cylinder connected via tube to a plastic bag. Postmortem examination in cases involving plastic bag-induced asphyxiation typically reveals nonspecific findings. Internal examination often shows visceral congestion, pulmonary and cerebral edema and the presence of unclotted blood in chambers of a heart. Additionally, characteristic signs of sudden death, such as petechial haemorrhages on the conjunctiva, epiglottis, heart and lungs surfaces are frequently observed. Those victims whose death was determined as a result of plastic bag-induced asphyxiation often use additional measures to achieve suicide such as medications like benzodiazepines and antidepressants, alongside ethyl alcohol consumption. Thus, toxicological analyses of victim’s blood and urine are necessity in detecting the exact concentrations of these substances within the body. Therefore, the integration of meticulous forensic investigation techniques, encompassing comprehensive evidence collection, detailed medical history review, thorough autopsy examination, and precise toxicological analyses, is paramount in unraveling the complexities surrounding plastic bag-induced asphyxiation cases, thereby advancing our understanding of these occurrences and enhancing forensic practices for future cases. In addition to these investigative measures, suicide prevention efforts for asphyxiations in confined spaces should focus on proactive surveillance, means restriction, and timely interventions, including limiting access to materials like compressed gases and mitigating the spread of information about novel suicide methods to reduce their adoption.
